# Reactive oxygen species-mediated apoptosis contributes to chemosensitization effect of saikosaponins on cisplatin-induced cytotoxicity in cancer cells

**DOI:** 10.1186/1756-9966-29-159

**Published:** 2010-12-09

**Authors:** Qiong Wang, Xue-lian Zheng, Lan Yang, Fang Shi, Lin-bo Gao, Ying-jia Zhong, Hong Sun, Fan He, Yong Lin, Xia Wang

**Affiliations:** 1 Laboratory of Molecular and Translational Medicine, West China Institute of Women and Children's Health, West China Second University Hospital, Sichuan University, Chengdu 610041, PR China; 2 Department of Forensic Biology, West China School of Preclinical and Forensic Medicine, Sichuan University, Chengdu 610041, PR China; 3 Department of Forensic Analytical Toxicology, West China School of Preclinical and Forensic Medicine, Sichuan University, Chengdu 610041, PR China

## Abstract

**Background:**

Saikosaponin-a and -d, two naturally occurring compounds derived from Bupleurum radix, have been shown to exert anti-cancer activity in several cancer cell lines. However, the effect of combination of saikosaponins with chemotherapeutic drugs has never been addressed. Thus, we investigated whether these two saikosaponins have chemosensitization effect on cisplatin-induced cancer cell cytotoxicity.

**Methods:**

Two cervical cancer cell lines, HeLa and Siha, an ovarian cancer cell line, SKOV3, and a non-small cell lung cancer cell line, A549, were treated with saikosaponins or cisplatin individually or in combination. Cell death was quantitatively detected by the release of lactate dehydrogenase (LDH) using a cytotoxicity detection kit. Cellular ROS was analyzed by flow cytometry. Apoptosis was evaluated by AO/EB staining, flow cytometry after Anexin V and PI staining, and Western blot for caspase activation. ROS scavengers and caspase inhibitor were used to determine the roles of ROS and apoptosis in the effects of saikosaponins on cisplatin-induced cell death.

**Results:**

Both saikosaponin-a and -d sensitized cancer cells to cisplatin-induced cell death in a dose-dependent manner, which was accompanied with induction of reactive oxygen species (ROS) accumulation. The dead cells showed typical apoptotic morphologies. Both early apoptotic and late apoptotic cells detected by flow cytometry were increased in saikosaponins and cisplatin cotreated cells, accompanied by activation of the caspase pathway. The pan-caspase inhibitor z-VAD and ROS scanvengers butylated hydroxyanisole (BHA) and N-acetyl-L-cysteine (NAC) dramatically suppressed the potentiated cytotoxicity achieved by combination of saikosaponin-a or -d and cisplatin.

**Conclusions:**

These results suggest that saikosaponins sensitize cancer cells to cisplatin through ROS-mediated apoptosis, and the combination of saikosaponins with cisplatin could be an effective therapeutic strategy.

## Background

Bupleurum radix, the dried root of Bupleurum falcatum, is one of the oldest and widely used crude drugs in traditional Chinese medicine. The major pharmaceutical ingredients in this plant are triterpene saponins, which include saikosaponin-a, -d, and -c. Among these compounds, saikosaponin-a (SSa) and saikosaponin-d (SSd) are the major active pharmacological components, which exert analgesic, anti-inflammatory, immunomodulatory, anti-viral, and hepatoprotective activities [1-4]. It is noteworthy that both SSa and SSd have been reported to induce cell cycle arrest and apoptosis in hepatoma cells, pancreatic cancer cells, breast cancer cells, and lung cancer cells [5-9], which makes them potential anti-cancer agents. Involvement of p53, nuclear factor kappaB and Fas/Fas ligand has been proposed for inhibition on cell growth and induction of apoptosis in human hepatoma cells by saikosaponin d [7]. However, the molecular mechanisms by which saikosaponins exert their anti-cancer effect are far from been elucidated.

Cisplatin (cis-diamminedichloroplatinum, DDP) is among the most effective and widely used chemotherapeutic agents employed for treatment of solid tumors. It is a platinum-based compound that forms intra- and inter-strand adducts with DNA, thus is a potent inducer of cell cycle arrest and apoptosis in most cancer cell types[10]. However, a major limitation of cisplatin chemotherapy is that many tumors either are inherently resistant or acquire resistance to the drug after an initial response. Multiple potential mechanisms of cisplatin chemoresistance have been proposed, including decrease of cellular concentration of the drug, enhancement of drug inactivation due to increased cellular levels of metallothionine and glutathione, increase of DNA repair, and alterations in signal pathways [10-13]. Tremendous efforts have been made to improve the anticancer value of cisplatin [14-17]. Naturally occurring compounds from diets or medicinal plants are good candidates for increasing cisplatin's anticancer activity [18,19]. The search for new compounds with high chemosensitization efficiency has never stopped.

Although several studies have shown that saikosaponins exert anti-cancer activity in several cancer cell lines, the effect of combining saikosaponins with chemotherapeutic drugs has never been addressed. In the present study, we found that both SSa and SSd, two major triterpene saponins could sensitize a number types of human cancer cells to cisplatin-induced cell death. Importantly, we found that the chemosensitization effect of saikosaponin is mainly mediated by the induction of cellular reactive oxygen species (ROS) accumulation in cancer cells. To our knowledge, this is the first report showing that saikosaponin-induced cellular ROS accumulation mediates synergistic cytotoxicity in saikosaponins and cisplatin co-treated cancer cells. These results suggest that saikosaponins are good adjuvant agents for sensitizing cancer cells to cisplatin, highlighting that the combination of saikosaponins and cisplatin could be an effective therapeutic strategy for improving the anticancer value of cisplatin.

## Materials and methods

### Reagents

Saikosaponin-a and -d were purchased from Chinese National Institute of the Control Pharmaceutical and Biological Products (Beijing, China). Cisplatin, Butylated hydroxyanisole (BHA) and N-acetyl-L-cysteine (NAC) were from Sigma (St. Louis, MO, USA). The pan-caspase inhibitor zVAD-fmk was purchased from Calbiochem (La Jolla, CA, USA). Antibodies against active caspase-3, poly (ADP-ribose) polymerase (PARP) were purchased from BD bioscience (San Diego, CA, USA). Anti-β-actin was purchased from Protein Tech (Chicago, IL, USA). 5-(and -6)-chloromethyl-2', 7'-dichlorodihydro-fluorescein diacetate acetyl ester (CM-H_2_DCFDA) and dihydroethidium (DHE) were purchased from Molecular Probes (Eugene, OR, USA).

### Cell culture

Two cervical cancer cell lines HeLa and Siha, an ovarian cancer cell line SKOV3, and a non-small cell lung cancer cell line A549 were from American Type Culture Collection (ATCC, Manassas, VA, USA) and grown in RPMI 1640 or DMEM supplemented with 10% fetal bovine serum (Hyclone, Thermo Scientific, Beijing, China), 1mmol/L glutamate, 100 units/mL penicillin, and 100 μg/mL streptomycin under standard incubator condition (37°C, 5% CO2).

### Cell death assay

Cells were seeded in 96-well plate one day before treatment and then treated as indicated in each figure legend. Cell death was assessed based on release of lactate dehydrogenase (LDH) using a cytotoxicity detection kit (Promega, Madison, WI, USA) as described previously [20]. All the experiments were repeated three to five times and the average is shown in each figure. For morphological study of cell death, cells were stained with 50 μg/mL of acridine orange and 50 μg/mL of ethidium bromide and then observed and photographed under a fluorescent microscope.

### Flow cytometry analysis after Anexin V and PI staining

Apoptosis was detected by flow cytometry using Annexin V-FITC Apoptosis Detection Kit (Nanjing KeyGen Biotech, Nanjing, China). Briefly, cells were double stained with annexin V-FITC and propidium iodide (PI) following manufacturer's instruction. Early apoptosis is defined by Annexin V^+^/PI^- ^staining (Q4) and late apoptosis is defined by Annexin V^+^/PI^+ ^staining (Q2) as determined by FACScan (Beckman coulter cell, Brea, CA, USA).

### Immunoblot analysis

Cells were treated as indicated in each figure legend and then cell extracts were prepared by lysing cells in M2 buffer [20 mmol/L Tris-HCl (pH 7.6), 0.5% NP40, 250 mmol/L NaCl, 3 mmol/L EDTA, 3 mmol/L EGTA, 2 mmol/L DTT, 0.5 mmol/L phenylmethylsulfonyl fluoride, 20 mmol/L β-glycerophosphate, 1 mmol/L sodium vanadate, and 1 μg/mL leupeptin]. Cell extracts were subjected to SDS-PAGE and analyzed by Western blot using various antibodies. The proteins were observed by enhanced chemiluminescence (Millipore, Billerica, MA, USA) using BIO-RAD Image station. Each experiment was repeated at least three times and representative results are shown in each figure.

### Detection of ROS

Cells cultured in 12-well plates were treated with saikosaponin or cisplatin alone or both as indicated in each figure legend. Cells were then stained for 30 minutes with 5 μM of H_2_O_2_-sensitive fluorescent dye CM-H_2_DCFDA or 5 μM of .O_2_^-^-sensitive dye dihydroethidium (DHE), washed 3 times with PBS, and subsequently assayed by FACScan (Beckman coulter cell, Brea, CA, USA) as reported previously [21].

### Statistical analysis

All numerical data are presented as mean ± standard deviation (SD) from at least three independent experiments. Statistical significance was analyzed by paired Student's t test using SPSS statistics software package and P < 0.05 was used for significance.

## Results

### Saikosaponin-a and -d sensitize cancer cells to cisplatin induced cytotoxicity

**Figure 1:**
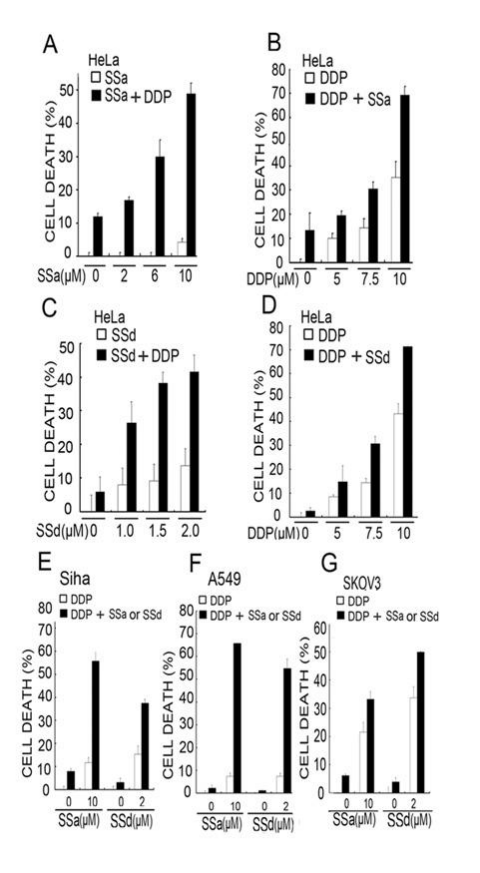
**Saikosaponin-a and -d sensitize cancer cells to cisplatin induced cytotoxicity**. (A) HeLa cells were treated with increasing concentrations of saikosaponin-a (2-10 μM) or fixed concentration of cisplatin (8 μM) alone or both for 48 hours. Cell death was measured by LDH release assay. Columns, mean of three experiments; bars, SD. (B) HeLa cells were treated with fixed concentration of saikosaponin-a (10 μM) or increasing concentrations of cisplatin (5-10 μM) alone or both for 48 h. Cell death was measured as described in (A). (C) HeLa cells were treated with increasing concentrations of saikosaponin-d or fixed concentration of cisplatin (8 μM) alone or both for 48 hours. Cell death was measured as described in (A). (D) HeLa cells were treated with fixed concentration of saikosaponin-d (2 μM) or increasing concentrations of cisplatin (5-10 μM) alone or both for 48 h. Cell death was measured as described in (A). (E), (F), (G) Siha cells, A549 cells, or SKOV3 cells were treated with cisplatin or 10 μM of saikosaponin-a or 2 μM of saikosaponin-d or combination of saikosaponin and cisplatin for 48 h. The dose of cisplatin is 30 μM for Siha, 8 μM for A549 and SKOV3, respectively. Cell death was measured as described in (A).

Both SSa and SSd have been reported to induce proliferation inhibition and cell death in various cancer cells (5-9). However, the effect of combination of these saikosaponins with chemotherapeutic drugs has never been investigated. We addressed this question by treating a cervical cancer cell line HeLa with SSa and cisplatin alone or both. Cell death was detected and quantified by an LDH release assay. While treatment with SSa alone caused marginal cell death (~10% cell death at 10 μM), it significantly sensitized cancer cells to cisplatin-induced cell death in a dose-dependent manner (~50% cell death at 10 μM concentration of SSa) (Figure 1A). A similar dose-dependent potentiation of cytotoxicity was observed with increasing cisplatin concentrations and a fixed SSa concentration (10 μM, Figure 1B). The potentiated effect could be detected with doses of SSa as low as 2 μM, a concentration of SSa by itself was nontoxic to the cells. Similar effect of SSd was detected in Hela cells, albeit SSd by itself is slightly more toxic than SSa (Figure 1C and 1D). The generality of potentiated cytotoxicity by combination of cisplatin with SSa or SSd was determined in another cervical cancer cell line Siha, an ovarian cancer cell line SKOV3, and a lung cancer cell line A549 treated under similar experimental conditions (Figure 1E, 1F, and 1G). These results suggest that both saikosaponin-a and -d could synergistically sensitize various cancer cells to cisplatin-induced cell death.

### Saikosaponins and cisplatin co-treatment potentiates apoptosis in cancer cells

**Figure 2:**
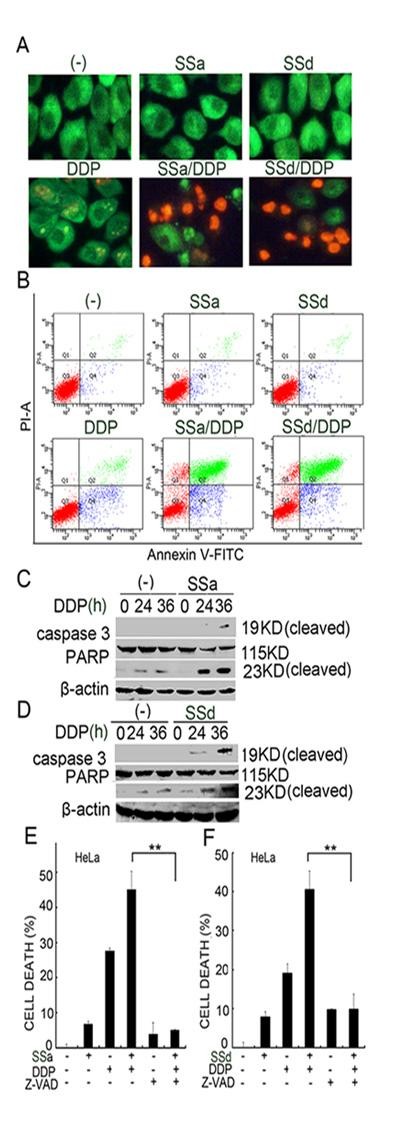
**Saikosaponins and cisplatin co-treatment potentiates apoptosis in cancer cells**. (A) HeLa cells were treated with cisplatin (8 μM) or saikosaponin-a (10 μM) or saikosaponin-d (2 μM) individually or combination of saikosaponin and cisplatin for 36 h and then stained with ethidium bromide and acridine orange; Cells were immediately observed and photographed under a fluorescence microscope. (B) HeLa cells were treated as indicated in (A), and then stained with annexin V and PI followed by flow cytometry analysis. Early apoptosis is defined by Annexin V^+^/PI^- ^staining (Q4) and late apoptosis is defined by Annexin V^+^/PI^+ ^staining (Q2). (C) and (D) HeLa cells were treated with cisplatin (8 μM) or saikosaponin-a (10 μM) or saikosaponin-d (2 μM) individually or combination of saikosaponin and cisplatin for 24 h and 36 h. Caspase -3 and PARP were detected by western blot. β-actin was detected as an input control. (E) and (F) HeLa cells were pretreated with zVAD-fmk (20 μM) for 30 min or remained untreated and then treated with saikosaponin-a or -d and cisplatin for another 48 h. Cell death was measured as described in Fig. 1A.

Cisplatin can induce two distinct modes of cell death, apoptosis and necrosis, in cancer cells [22,23]. Saikosaponins were also reported to activate apoptosis in hepatoma cells [7]. To determine the mode of cell death induced by saikosaponin and cisplatin co-treatment, we first detect morphological changes in saikosaponin and cisplatin-cotreated HeLa cells by acridine orange/ethidium bromide staining followed by fluorescent microscopy. As shown in Figure 2A, typical apoptotic features such as cell shrinkage, cell membrane blebbing, and nuclear condensation were observed microscopically in cotreated cells. Consistently, both early apoptotic and late apoptotic cells as determined by flow cytometry after annexin V and PI staining were significantly increased when the cells were treated with the combination of saikosaponin-a or -d and cisplatin (Figure 2B). Western blot revealed that activation of caspase 3 was potentiated in the co-treated HeLa cells (Figure 2C and 2D). In addition, the cleavage of the caspase-3 substrate PARP (115 kDa) and generation of the small fragment (23-kDa) in the co-treated cells were also significantly enhanced (Figure 2C and 2D). Furthemore, the pan-caspase inhibitor zVAD-fmk significantly suppressed the synergistic cytotoxicity induced by co-treatment with SSa or SSd and cisplatin (Figure 2E and 2F). Collectively, these results suggest that apoptosis is involved in the potentiation of cytotoxicity caused by saikosaponins and cisplatin co-treatment.

### Saikosaponins induce intracellular ROS accumulation in cancer cells

**Figure 3:**
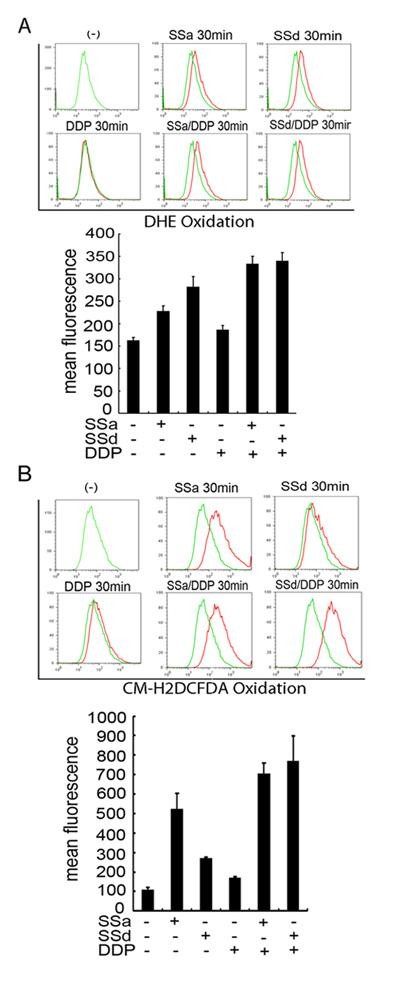
**Saikosaponins induce intracellular ROS accumulation in HeLa cells**. HeLa cells were treated with cisplatin (8 μM) or saikosaponin-a (10 μM) or saikosaponin-d (2 μM) individually or combination of saikosaponin and cisplatin for 30 min. 5 μM of DHE (A) or 5 μM of CM-H_2_DCFDA (B) was added 30 min before collecting cells. The fluorescent intensities of 10,000 cells were analyzed with a flow cytometer. Untreated cells with DHE or CM-H_2_DCDA staining were used as a negative control. The histogram overlays show the results of treated cells (red lines) compared with untreated cells (green lines). x-axis, fluorescent intensity showing the extent of DHE or CM-H_2_DCFDA oxidation; y-axis, cell number. The data (mean fluorescence for each group) was also presented as bar charts below the profiles (error bars indicate SD of triplicate experiments).

ROS such as superoxide anion (.O_2_^-^) and its reduced product hydrogen peroxide (H_2_O_2_) have been considered as cytotoxic byproducts of cellular metabolism, and the accumulation of ROS in cells may promote cell death. Although saikosaponins have been reported to be antioxidants that improve hepatic antioxidant capacity and protects against CCl_4_-induced liver injury in rats [24], their roles in intracellular redox modulation have never been addressed. To investigate the mechanism of the saikosaponins and cisplatin-induced cytotoxicity, we examined the effect of saikosaponin and cisplatin on ROS levels in HeLa cells. Cells treated with saikosaponin, cisplatin, or both were stained with two ROS-specific dyes, CM-H_2_DCFDA that is specific for hydrogen peroxide (H_2_O_2_) or DHE that is specific for .O_2_^-^. Cisplatin had marginal effect on cellular .O_2_^- ^level. Whereas, either SSa or SSd strongly induced cellular .O_2_^- ^accumulation (Figure 3A, rightward shift of the peaks). The treatment with SSa or SSd plus cisplatin retained similar trend of .O_2_^- ^induction as treated by the saikosaponins alone. Similar trend and more striking extent of H_2_O_2 _induction by SSa or SSd, alone or in combination with cisplatin were observed (Figure 3B). Notably, the induction of ROS by saikosaponins was also detected in Siha, A549, and SKOV3 cells (Additional file 1 Fig. S1), suggesting that the modulation of cellular redox status by saikosaponins is a common effect in cancer cells that we tested. Altogether, these results indicate that cellular ROS were strongly induced by SSa or SSd, suggesting that both these saikosaponins function as pro-oxidants in cancer cells.

### ROS accumulation contributes to the synergistic cytotoxicity induced by saikosaponins plus cisplatin

**Figure 4:**
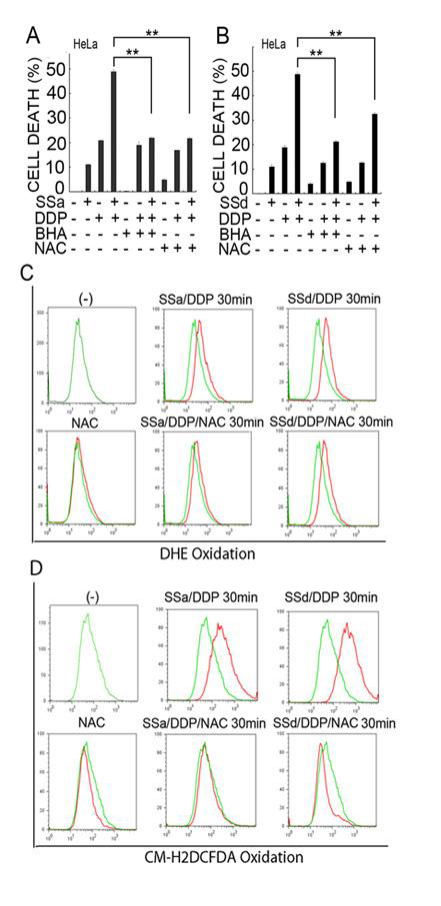
**ROS accumulation contributes to the synergistic cytotoxicity induced by saikosaponins plus cisplatin in HeLa cells**. (A) and (B) HeLa cells were pretreated with BHA (100 μM) or NAC (1 mM) for 30 min or remained untreated and then treated with saikosaponin-a (10 μM) or saikosaponin-d (2 μM) or cisplatin (8 μM) individually or combination of saikosaponin and cisplatin for 48 h. Cell death was measured as described in Fig. 1A. (C) and (D) HeLa cells were pretreated with NAC (1 mM) for 30 min or remained untreated and then treated with saikosaponin-a (10 μM) or saikosaponin-d (2 μM) or cisplatin (8 μM) alone or combination of saikosaponin and cisplatin for another 30 min. Cells were stained with DHE (C) or CM-H_2_DCFDA (D) 30 min before collecting cells and then analyzed by flow cytometer.

**Figure 5:**
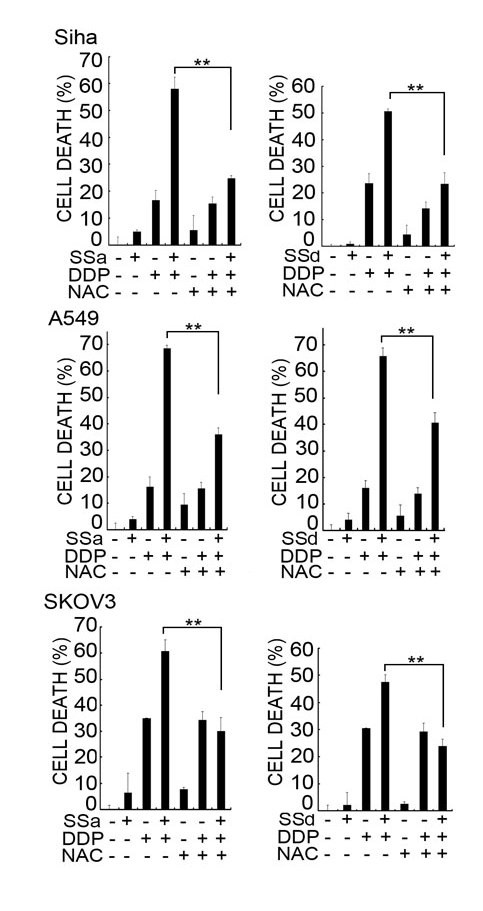
**ROS accumulation contributes to the synergistic cytotoxicity induced by saikosaponins plus cisplatin in Siha cells, A549 cells, and SKOV3 cells**. Siha cells (A), A549 cells (B), and SKOV3 cells (C) were pretreated with NAC (1 mM) for 30 min or remained untreated and then treated with saikosaponin-a (10 μM) or saikosaponin-d (2 μM) or cisplatin individually or combination of saikosaponin and cisplatin for 48 h. The dose of cisplatin is 30 μM for Siha, 8 μM for A549 and SKOV3, respectively. Cell death was measured as described in Fig. 1A.

We next investigated whether the ROS accumulation is required for the potentiated cytotoxicity induced by saikosaponins and cisplatin co-treatment. As shown in Figure 4A, both the ROS scavengers BHA and NAC almost completely suppressed the potentiation of cisplatin-indcued cytotoxicity by SSa. Similarly, the ROS scanvengers also effectively inhibited the enhanced cell death in SSd and cisplatin cotreated cells (Figure 4B). The inhibition effect of ROS scavengers on cell death was correlated with significant reduction of .O_2_^- ^and H_2_O_2 _levels in cells (Figure 4C and 4D). To further confirm the effect of ROS in synergistic cytotoxicity induced by saikosaponins plus cisplatin, Siha, A549, and SKOV3 cells were pretreated with NAC and then treated with saikosaponins and cisplatin individually or both. As expected, NAC also suppressed the enhanced cell death mediated by saikosaponins and cisplatin co-treatment in these cells (Figure 5A, 5B, and 5C). These results suggest that induction of ROS is crucial for saikosaponins' potentiation effect on cisplatin-induced cytotoxicity in cancer cells.

## Discussion

In this study we demonstrated that both SSa and SSd potently sensitize a number of human cancer cells to cisplatin-induced apoptosis through ROS accumulation. First, the chemosensitization effect of SSa and SSd appeared to be general in solid cancer cells, including those derived from cervix, ovary, and lung. Second, the enhanced cell death in saikosaponin and cisplatin-cotreated cells was mainly apoptotic because the co-treated cells showed typical apoptotic morphology, increased early apopototic and late apoptotic cell population, and activation of caspases. Furthermore, the chemosensitization effect of saikosaponins could be efficiently blocked by the pan-caspase inhibitor zVAD-fmk. Third, both SSa and SSd induced .O_2_^- ^and H_2_O_2 _accumulation in cancer cells and pretreatment of cells with ROS scavengers effectively inhibited the potentiated cytotoxicity. To our knowledge, this is the first report showing that saikosaponins sensitize cisplatin-induced cell death through modulation of redox status in cancer cells. The combination of saikosaponins and cisplatin could greatly improve the sensitivity of cancer cells to cisplatin.

Combination with agents that sensitize cancer cell to chemotherapeutics has been recognized as an efficient strategy to overcome chemoresistance. Naturally occurring compounds from diets or medicinal plants are generally safe and associated with low toxicity, making them ideal candidates for increasing anticancer drugs' activity. Saikosaponin-a and -d, two major triterpene saponins derived from Bupleurum radix, have been reported previously to have anticancer property [6,8]. However, the effect of combination of saikosaponins and chemotherapeutics has never been addressed. In the present study we found that non-toxic dose of either SSa or SSd could sensitize a panel of cancer cells to cisplatin-induced cell death. It is unlikely that p53 is involved in the synergistic cytotoxicity of saikosaponins and cisplatin, because this anticancer effect was detected in cancer cell lines with both wild-type p53 (A549), inactivated p53 (HeLa) and mutated p53 (SKOV3). Indeed, the independence of p53 would be an advantage of this combination for cancer therapy because p53 is mutated in many types of tumors. The sensitization effect of saikosaponin was mainly through enhancing the cisplatin-induced apoptosis, which was accompanied by enhanced activation of caspase 3 and the cleavage of caspase 3 substrate PARP, and was blocked by the caspase inhibitor z-VAD. It is noteworthy that Siha cell, which is a well known cervical cancer cell line resistant to cisplatin, was significantly sensitized to cisplatin-induced cell death, suggesting that saikosaponins are potent adjuvant that are able to override primary cisplatin resistance in cancer. Thus, results from this study reveal a novel function of saikosaponins that adds up the anticancer value of these naturally occurring compounds.

Many naturally occurring compounds have been reported to exert anti-cancer effect through ROS induction. For example, d-Limonene, a bioactive food component from citrus, was found to augments the cytotoxic effects of docetaxel through induction of cellular H_2_O_2 _[25]. Our finding in this study also showed that both SSa and SSd induced significant cellular ROS accumulation in cancer cells, which substantially contribute to synergistic cytotoxicity in saikosaponin and cisplatin cotreated cell. It was previously found that saikosaponins exhibit antioxidant activity in normal hepatocytes [24]. The reason of discrepancy is currently unclear, but could be explained by differences in cellular contents. Indeed, redox regulating compounds such as flavonoid luteolin can function as an antioxidant in normal cells while as a pro-oxidant in cancer cells [26]. It remains to be determined that how distinct redox modulating functions are executed in normal and cancerous condition.

## Conclusion

Our results suggest that saikosaponin-a and -d are potent in sensitizing cancer cells to cisplatin-induced apoptosis through ROS accumulation. Thus, the combination of saikosaponins with cisplatin could increase the therapeutic effect of cisplatin against solid tumors.

## Competing interests

The authors declare that they have no competing interests.

## Authors' contributions

XW and YL designed research and wrote and revised the manuscript; QW performed all research experiments and analyzed data; XLZ assisted with cell death experiment. LY and YJZ assisted with flow cytometry experiment; FS, LBG, HS and FH assisted with cell culture and immunoblots. All authors read and approved the final manuscript.

## Supplementary Material

Additional file 1 **Figure S1**. Saikosaponins induce intracellular ROS accumulation in Siha cells, A549 cells, and SKOV3 cells. Siha cells, A549 cells, and SKOV3 cells were treated with saikosaponin-a (10 μM) or saikosaponin-d (2 μM) for 30 min respectively and stained with 5 μM of CM-H_2_DCFDA. The fluorescent intensities were detected by flow cytometry. Click here for file
