# Supplementary figures and images for: Reactive oxygen species-mediated apoptosis contributes to chemosensitization effect of saikosaponins on cisplatin-induced cytotoxicity in cancer cells
Source: J Exp Clin Cancer Res. 2010 Dec 9;29(1):159. doi: 10.1186/1756-9966-29-159 (PMC3006358; doi:10.1186/1756-9966-29-159)

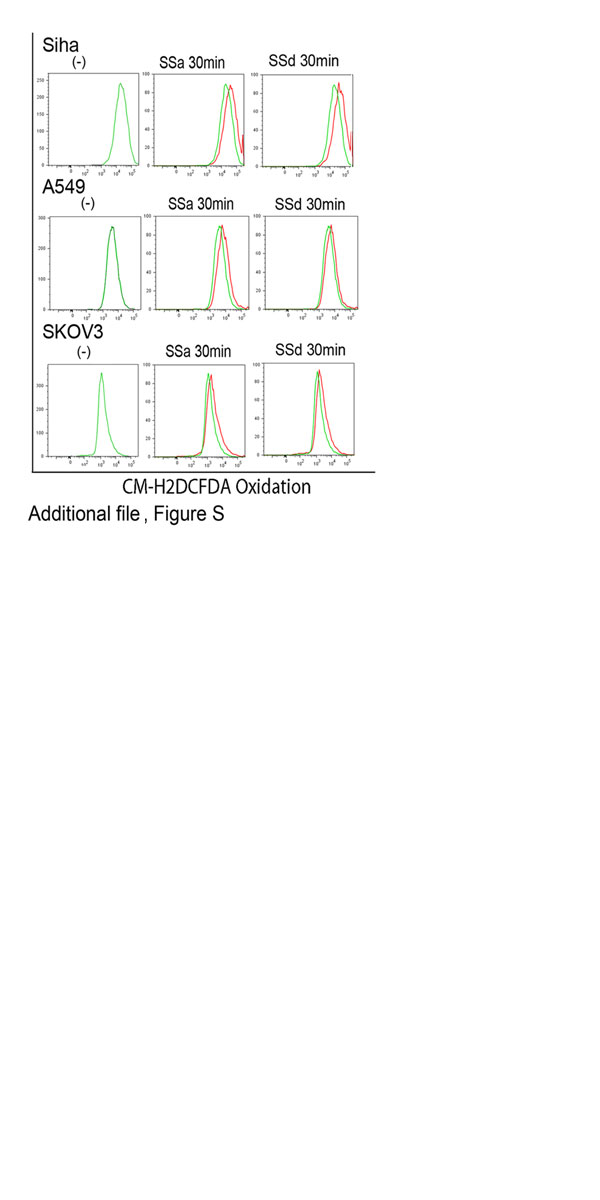

Supplement: Additional file 1 — Figure S1. Saikosaponins induce intracellular ROS accumulation in Siha cells, A549 cells, and SKOV3 cells. Siha cells, A549 cells, and SKOV3 cells were treated with saikosaponin-a (10 μM) or saikosaponin-d (2 μM) for 30 min respectively and stained with 5 μM of CM-H2DCFDA. The fluorescent intensities were detected by flow cytometry. [file 1756-9966-29-159-S1.JPEG]
